# Calculating the similarity between prescriptions to find their new indications based on graph neural network

**DOI:** 10.1186/s13020-024-00994-y

**Published:** 2024-09-11

**Authors:** Xingxing Han, Xiaoxia Xie, Ranran Zhao, Yu Li, Pengzhen Ma, Huan Li, Fengming Chen, Yufeng Zhao, Zhishu Tang

**Affiliations:** 1 State Key Laboratory for Quality Ensurance and Sustainable Use of Dao-Di Herbs, National Resource Center for Chinese Materia Medica, China Academy of Chinese Medical Sciences, Beijing, 100700 People’s Republic of China; 2 National Data Center of Traditional Chinese Medicine, China Academy of Chinese Medical Sciences, Beijing, 100700 People’s Republic of China; 3 Beijing University of Chinese Medicine, Beijing, 100029 People’s Republic of China

**Keywords:** Similarity between prescriptions, New indications, Graph neural network

## Abstract

**Background:**

Drug repositioning has the potential to reduce costs and accelerate the rate of drug development, with highly promising applications. Currently, the development of artificial intelligence has provided the field with fast and efficient computing power. Nevertheless, the repositioning of traditional Chinese medicine (TCM) is still in its infancy, and the establishment of a reasonable and effective research method is a pressing issue that requires urgent attention. The use of graph neural network (GNN) to compute the similarity between TCM prescriptions to develop a method for finding their new indications is an innovative attempt.

**Methods:**

This paper focused on traditional Chinese medicine prescriptions containing ephedra, with 20 prescriptions for treating external cough and asthma taken as target prescriptions. The remaining 67 prescriptions containing ephedra were taken as to-be-matched prescriptions. Furthermore, a multitude of data pertaining to the prescriptions, including diseases, disease targets, symptoms, and various types of information on herbs, was gathered from a diverse array of literature sources, such as Chinese medicine databases. Then, cosine similarity and Jaccard coefficient were calculated to characterize the similarity between prescriptions using graph convolutional network (GCN) with a self-supervised learning method, such as deep graph infomax (DGI).

**Results:**

A total of 1340 values were obtained for each of the two calculation indicators. A total of 68 prescription pairs were identified after screening with 0.77 as the threshold for cosine similarity. Following the removal of false positive results, 12 prescription pairs were deemed to have further research value. A total of 5 prescription pairs were screened using a threshold of 0.50 for the Jaccard coefficient. However, the specific results did not exhibit significant value for further use, which may be attributed to the excessive variety of information in the dataset.

**Conclusions:**

The proposed method can provide reference for finding new indications of target prescriptions by quantifying the similarity between prescriptions. It is expected to offer new insights for developing a scientific and systematic research methodology for traditional Chinese medicine repositioning.

**Supplementary Information:**

The online version contains supplementary material available at 10.1186/s13020-024-00994-y.

## Introduction

Expanding new indications, which aims to use existing drugs or drug candidates for diseases different from the original therapeutic range, is a specific application practice in the field of drug repositioning. Drug repositioning (also known as drug repurposing) is a strategy for identifying new uses for drugs that have been approved or are under investigation. Obviously, this strategy can shorten the drug development time and save costs, and the risk of failure in late-stage clinical trials is lower because the material basis and safety studies have been guaranteed [1]. The average cost of a new drug discovery and development process is estimated to range from $1 to $2 billion, with an average development time of approximately 15 years [2]. Consequently, drug repositioning, a more cost-effective and efficient drug discovery strategy, has attracted considerable attention from researchers [3]. For instance, minoxidil, initially employed to treat hypertension, has been demonstrated to be efficacious in the treatment of alopecia [4]. Furthermore, zidovudine, which was previously employed as an anti-tumor agent, became the inaugural anti-AIDS medication to be approved for use [5].

Currently, the repositioning research of chemical drugs has achieved significant results, whereas that of traditional Chinese medicine (TCM)remains in its infancy and should be emphasized. TCM is an important resource treasure trove of the Chinese nation. Moreover, constantly evolving modern technology utilized for repositioning research can help alleviate the limitations of TCM applications and further explore and expand its clinical value. At the theoretical level, TCM has a long history of application and a rich theoretical system. Modern research has also demonstrated that TCM is characterized by multi-components, multi-targets, and multi-pathways, which provides a theoretical basis for conducting TCM repositioning studies [6]. Simultaneously, in the new era of medical advancement, abundant measurement data can be obtained through sophisticated analytical tools, while faster computational efficiency can be achieved by the rapid progress of information technology [7, 8]. On a practical level, China's large population and complex disease spectrum provide realistic conditions for repositioning research in TCM. Since the outbreak of COVID-19, the research on TCM prescriptions to combat COVID-19 has promoted the development of repositioning of TCM [9, 10]. A total of 35 varieties of TCM prescriptions have been entered into the registration and acceptance of medicines in the form of adding functions and indications. For example, the Jingfang mixture from Lunan Houpu Pharmaceutical Co., Ltd. was initially intended for the treatment of colds. However, recent repositioning research has discovered its potential use in treating skin ailments such as urticaria and itching [11, 12].

Existing research ideas on TCM repositioning can be broadly categorized into two groups [13]. One is to try existing medications for new diseases or symptoms. The other is to generalize new uses based on the clinical applications of existing drugs, that is, using human experience. Regardless of the repositioning research strategy chosen, the pivotal challenge lies in establishing the correlation between drugs and diseases, whether from a macro or micro perspective.

Considering the multi-target and multi-pathways characteristics of TCM, network pharmacology plays a crucial role in the development of TCM repositioning. By constructing a network of “disease-target- ingredient-TCM” interactions, it can analyze the mechanism of action of TCM, making it easier to discover new targets and thereby enhancing the success rate of TCM repositioning [14, 15]. For example, Yinying Wang et al. introduced a new network pharmacology approach to predict potential novel indications of old drugs based on the molecular networks affected by drugs and associated with disease, using Liuwei Dihuang Wan as a case study. And the prediction outcomes were corroborated by expert insights and supporting evidence from existing literature [16]. Molecular docking is another method employed to calculate drug-disease associations. This involves the identification of new targets through the selection of target proteins from a database that can bind to a given ligand [17].

In addition to the micro information, such as ingredients and targets above-mentioned, macro information, including real-world clinical observations, is also of equal importance. Thus, more efficient computational methods are urgently needed to better compute the relationships between massive and multi-type data. With the development of artificial intelligence, traditional machine learning or the newly emerging deep learning provide new screening methods for drug repositioning [18]. Eunyoung Kim et al. used machine learning methods, including logistic regression, random forests, and support vector machine algorithms, to calculate four categories of drug-drug similarity (e.g., chemical structure, side-effects, gene ontology, and targets) and three categories of disease-disease similarity (e.g., phenotypes, human phenotype ontology, and gene ontology). The results indicated that the model could contribute to drug discovery by suggesting drug candidates from herbal compounds which have potentials but few were studied [19].

Nevertheless, compared to other chemical repositioning, the application of deep learning in TCM repositioning has been less studied. Among various learning methods, graph neural network (GNN) has been the most widely employed. Some articles demonstrated that GNN can be used to construct and learn information graphs of drugs and diseases, as well as their correlations, to predict the probability of interactions between each disease-drug pair [20, 21].

In light of the current state of research on the repositioning of Chinese medicines, it is evident that certain elements remain to be added. Therefore, the main work and contributions of this paper are as follows. Firstly, Chinese patent drugs were selected as the research objects, as they are listed prescriptions and have a clear range of indications. Combining our team’s previous research, it was ultimately decided to take Chinese patent drugs, which contain ephedra and are mainly used to treat cough and asthma caused by external factors, as an example. Secondly, most of the data currently being used are measurement information such as composition and targets, lacking integration with classical TCM literature. Therefore, a comprehensive dataset integrating the application of ancient classic prescriptions and molecular information data can be constructed as the data foundation for this paper. Thirdly, GNN was used to calculate the similarity of network graphs of prescriptions, herbs symptoms, ingredients, and targets that incorporate different types of data since it is more suitable for expressing multi-dimensional information about prescriptions. Finally, potential new indications for a given prescription were sought by comparing the similarity between prescriptions using two metrics: cosine similarity and Jaccard coefficient.

## Materials and methods

### Collection of information on target prescriptions

- (1) Based on the therapeutic information of the prescriptions, Chinese patent drugs for the treatment of external cough and asthma were screened by the Chinese Pharmacopoeia 2020. The target prescriptions were selected from them using “ephedra” as a filtering term.
- (2) Extract information on the symptoms, herbs, categories, properties, flavors, and meridians of herbs of the target prescriptions were obtained from the Chinese Pharmacopoeia.
- (3) The ingredients and target proteins of herbs were obtained from well-known databases such as TCMSP (https://www.tcmsp-e.com/#/home) and SymMap (http://www.symmap.org/).
- (4) The target protein names in (3) were converted to gene names using the Uniprot(https://www.uniprot.org/) database, and the original protein names were retained if they could not be matched.
- (5) Disease information related to the target prescriptions was obtained from the existing literature reports, and the disease names were standardized according to the China Medicine Information Query Platform (https://www.dayi.org.cn). Furthermore, we searched and downloaded pertinent target information from databases like OMIM and GeneCards based on disease names, eliminated duplicate gene names, and then saved them.

### Collection of information on prescriptions to be matched

- (1) prescriptions containing ephedra were extracted from the following sources: “Shanghan Zabing Lun”, “The First Batch of the Catalog of Ancient Classical Famous Prescriptions”, and “The Second Batch of the Catalog of Ancient Classical Famous Prescriptions”. The prescriptions for treating coughs and asthma caused by external factors were then removed, resulting in the prescriptions to be matched.
- (2) The collected symptom names were standardized and summarized according to the “Code of Terminology for Common Clinical Symptoms in TCM”, and the original names of the symptoms were retained if they were not categorized for the time being. The collected herb names were standardized and summarized according to the Chinese Pharmacopoeia, and if they were not included, their original names were retained.
- (3) Extract the information of the herbs mentioned above from the Chinese Pharmacopoeia, such as their categories, properties, flavors, and meridians.
- (4) The next steps were consistent with those described in paragraphs (3), (4), and (5) of the section titled 'Collection of Information on Target Descriptions'.

### Calculating cosine similarity and Jaccard coefficient based on GCN

GNN represents a subfield of deep learning designed to learn graph-structured data. This enables the extraction and uncovering of features and patterns within the data, thus meeting the needs of graph learning tasks, including clustering, classification, prediction, segmentation, and generation [22]. In this paper, the data on traditional Chinese medicine prescriptions used and the relationships between them can essentially be regarded as graph-structured data, which is very suitable for processing using graph-embedding learning methods. Moreover, the data selected does not have clear labels, making it more suitable for processing with self-supervised learning methods. In light of the aforementioned considerations, the self-supervised learning method DGI (DEEP GRAPH INFOMAX) proposed by Petar Veličković et al. was referenced as the experimental method [23]. The objective of DGI is to identify the optimal node representations in a graph by maximizing the mutual information between the node embedding representation and the global graph embedding representation. This approach does not necessitate the labeling of nodes or the specification of a particular graph structure; rather, it utilizes the structural information present in the graph to learn the embedding vectors of nodes.

Furthermore, prescriptions consist of multiple herbs with distinct attributes, which can be considered as a complex system with multi-dimensional information. Consequently, quantifying the similarity of prescriptions poses a fundamental research challenge in the field of TCM data mining [24, 25]. After comparing existing quantitative methods, the paper decided to use two metrics, cosine similarity and Jaccard coefficient, to characterize the similarity between prescriptions. In this paper, cosine similarity is evaluated by calculating the cosine value of the angle between two vectors, with a value range of [− 1,1]. The greater the value, the greater the degree of similarity. Jaccard coefficient treats a prescription as a collection of herbs. Its calculation formula is the ratio of the intersection of two sets to the union of the two sets, with a value range of [0,1]. The greater the value, the greater the degree of similarity.

In particular, all nodes, including those pertaining to prescriptions, diseases, symptoms, herbs, and so forth, are selected to calculate the cosine similarity between them. With regard to the Jaccard coefficient, the nodes chosen for calculation are prescriptions, diseases, symptoms, herbs, and their attributes. Given the calculation principle of this indicator, it is not necessary to include all molecular information nodes.

**Fig. 1:**
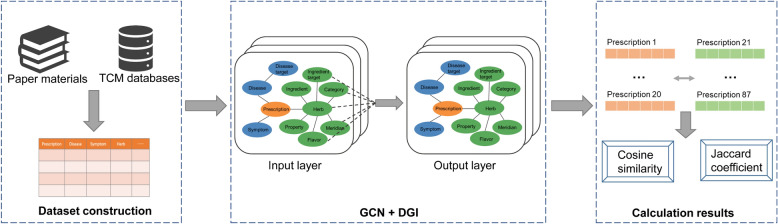
The framework of the methodology

**Fig. 2:**
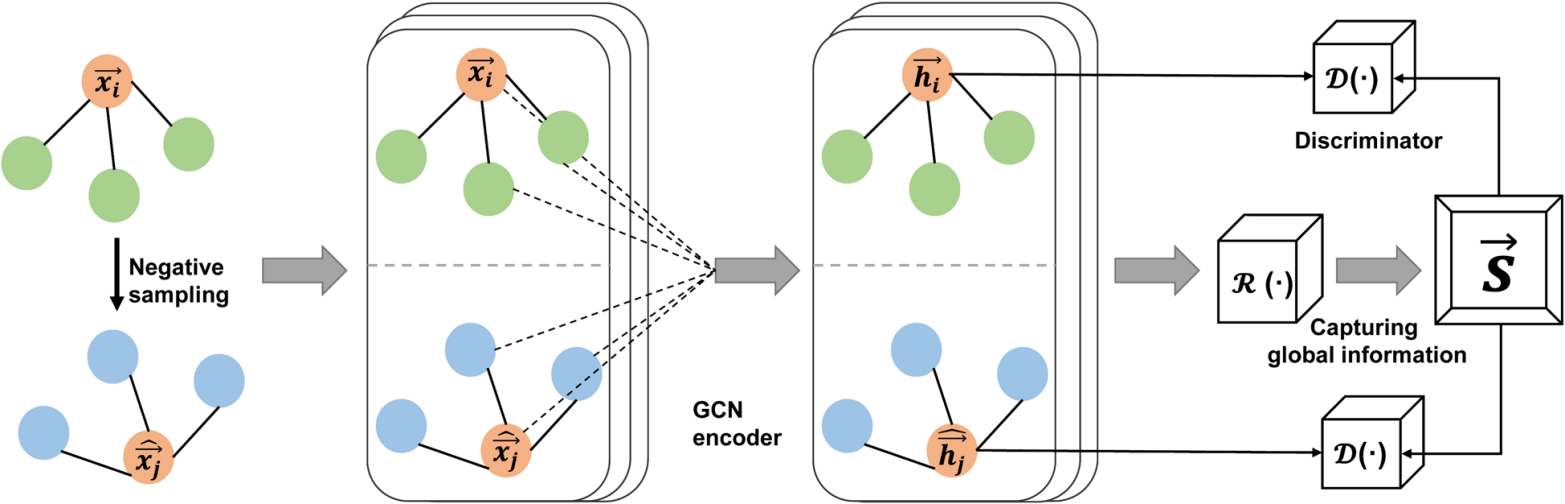
The schematic of the model

In consideration of the aforementioned factors, the running structure of the model presented in this paper is as follows: Firstly, GCN is employed to receive graph-structured data containing all information and to learn the embedding representation of each node, thereby outputting the feature vectors of each node. Subsequently, DGI is used to further optimize these embedding representations, aiming to maximize the mutual information between the node embeddings and the global graph embeddings. Finally, the optimized feature vectors are employed to compute the similarity between the nodes of the prescriptions. The framework of the methodology of this paper is shown in Fig. 1, while the schematic of the model is shown in Fig. 2. The running process of the model is as follows.

- (1) Preparation of positive and negative samples

Positive samples: a set of node features is provided, $X$ = {$\overrightarrow{{x}_{1} },\overrightarrow{{x}_{2} },\dots , \overrightarrow{{x}_{N}}$}, where $N$ is the number of nodes and $\overrightarrow{{x}_{i}}$ represents the features of node$i$. The adjacency matrix is defined as$A$. In this paper, the graphs are assumed to be unweighted, i. e., ${A}_{ij}$ = 1 if there exists an edge $i$→$j$ in the graph and ${A}_{ij}$ = 0 otherwise. Negative samples: an explicit corruption function C is used to perturb the graph by randomly upsetting the node features. The process can be expressed as ($\widehat{X }, \widehat{A}$) = C(*X, A*), $\widehat{X}$ = {$\overrightarrow{{x}_{1} },\overrightarrow{{x}_{2} },\dots , \overrightarrow{{x}_{M}}$}, where $M$ is the number of nodes and $\widehat{A}$ is the adjacency matrix.

- (2) Embedding of nodes

Define $\varepsilon (X, A)$ = H = {$\overrightarrow{{h}_{1} },\overrightarrow{{h}_{2} },\dots , \overrightarrow{{h}_{N}}$}, where H represents high-level representations. The specific formula is (1).

1 $${H}^{\left(l+1\right)}=\sigma \left({\widetilde{D}}^{-1/2}\widetilde{A}{\widetilde{D}}^{-1/2}{H}^{\left(l\right)}{W}^{\left(l\right)}\right)$$

where ${H}^{\left(l+1\right)}$ is the node features of layer $l+1$, $\sigma$ is a nonlinear activation function, $\widetilde{A}$ is a normalized adjacency matrix including self-connections, $\widetilde{D}$ is the degree matrix of $\widetilde{A}$, ${W}^{\left(l\right)}$ is a learnable weight parameter for layer $l$.

- (3) Graph-level summary representation

A readout function $\mathcal{R}$ is used to summarize the obtained patch representations into a graph-level representation, i. e., $\overrightarrow{s}$ = $\mathcal{R}\left(\varepsilon \left(X, A\right)\right).$ The specific formula is (2).

2 $$\mathcal{R}\left(H\right)= \sigma (\frac{1}{N}\sum_{i=1}^{N}\overrightarrow{{h}_{i} })$$

- (4) Maximizing mutual information

A discriminator D is used to distinguish between true patch-summary pairs (positive samples) and false patch-summary pairs (negative samples), which can represent the probability scores assigned to this patch-summary pair. The specific formula is (3).

3 $$\mathcal{D}\left(\overrightarrow{{h}_{i} }, \overrightarrow{s }\right)= \sigma ({\overrightarrow{{h}_{i} }}^{T}W\overrightarrow{s })$$

The standard binary cross-entropy loss function is employed as the objective function, which measures the difference between positive and negative samples, to maximize the mutual information between $\overrightarrow{{h}_{i}}$ and $\overrightarrow{s}$ while minimizing the mutual information between $\overrightarrow{\widehat{{h}_{i}}}$ and $\overrightarrow{s}$. The specific formula is (4).

4 $$\mathcal{L}= \frac{1}{N+M}(\sum_{i=1}^{N}{\mathbb{E}}_{\left(X, A\right)}\left[log\mathcal{D}\left(\overrightarrow{{h}_{i} }, \overrightarrow{s }\right)\right]+\sum_{j=1}^{M}{\mathbb{E}}_{\left(\widehat{X }, \widehat{A }\right)}\left[\text{log}(1-\mathcal{D}\left(\overrightarrow{\widehat{{h}_{i}}}, \overrightarrow{s}\right))\right])$$

## Results

### Presentation of the dataset

In this paper, a total of 20 target prescriptions and 67 prescriptions to be matched were collected. In this case, there were three dimensions of information for each prescription, as shown in Table 1. The first dimension was the name of the prescriptions. The second dimension was information on diseases, including diseases, symptoms, and disease targets. The third dimension is information on herbs, including herbs, their categories, properties, flavors, meridians, ingredients, and ingredient targets.

**Table 1:** Information of the dataset

	Type of nodes	Number of nodes
The first dimension	Prescription	87
The second dimension	Disease	84
	Symptom	113
	Disease target	10,856
The third dimension	Herb	183
	Property	5
	Flavor	12
	Meridian	12
	Category	35
	Ingredient	1294
	Ingredient target	2529

### Cosine similarity calculation results

**Fig. 3:**
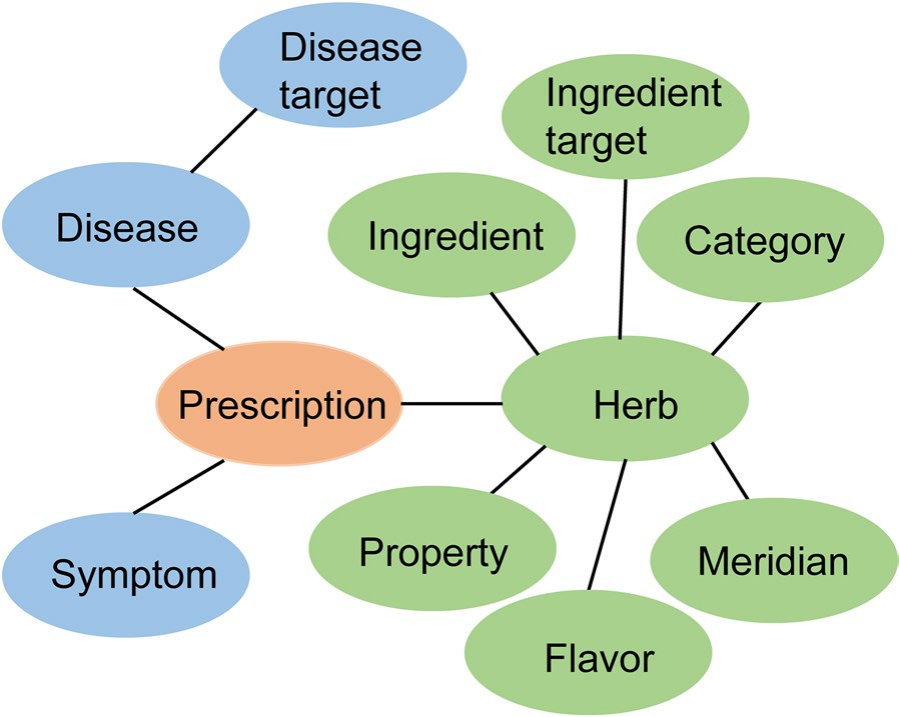
Graph structure data of prescriptions containing multidimensional information

**Fig. 4:**
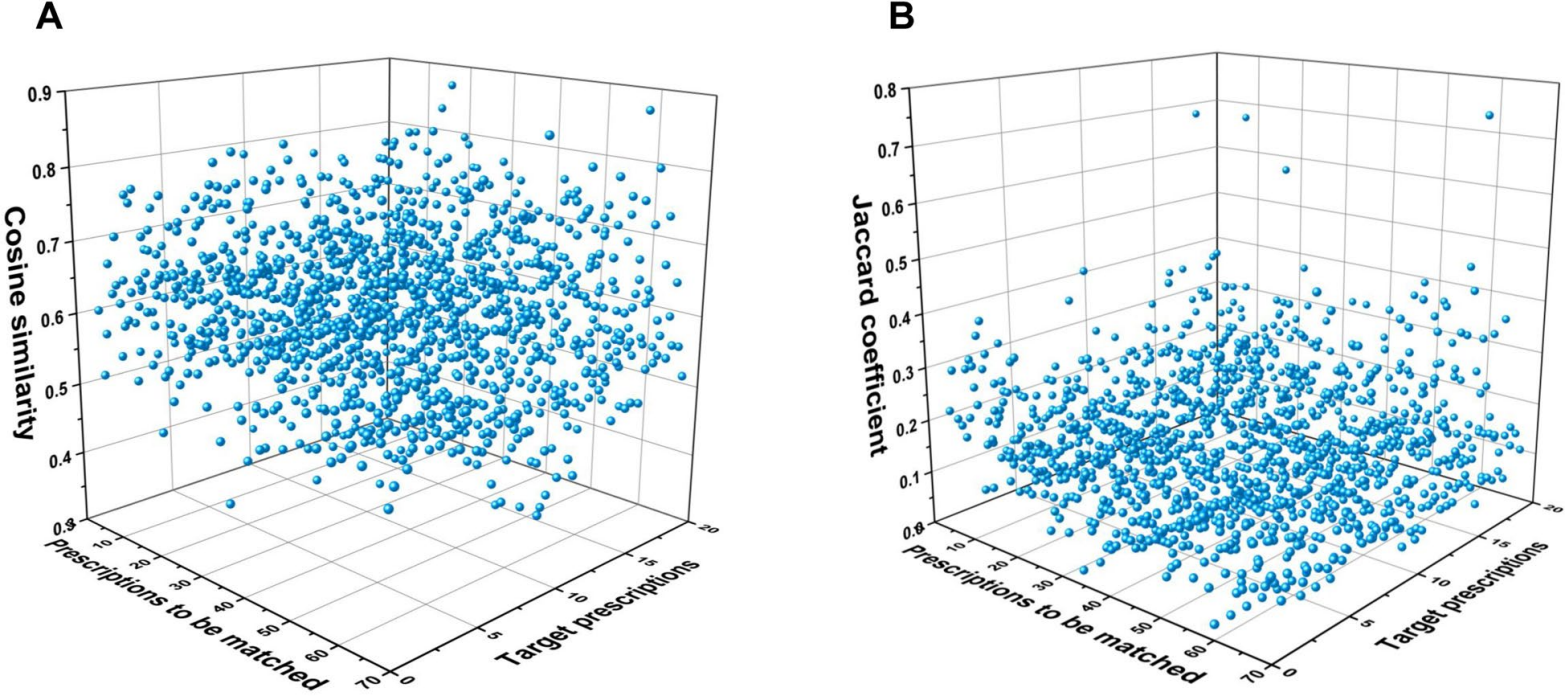
3D schematic diagram of cosine similarity raw data (**A**) and Jaccard coefficient raw data (**B**)

The information in the dataset was constructed into graph-structured data, as shown in Fig. 3, and calculated. Due to the extensive number of calculation results that could not be fully displayed, only the data processing results are presented in Table 2. Figure 4 presented a three-dimensional schematic of the original data. The combination of the two charts can reveal the distribution of the data. Please refer to the supplementary material for access to all raw data.

**Table 2:** Analysis of the results of the two calculated metrics

	Average value	Maximum value	Minimum value	Standard deviation	Kurtosis	Skewness	P25	P75	P95
Cosine similarity	0.607	0.88	0.25	0.0930	− 0.181	0.012	0.54	0.67	0.77
Jaccard coefficient	0.140	0.73	0	0.0923	3.312	1.356	0.07	0.19	0.32

The results indicated that the average of 1340 cosine similarities was 0.607, with a standard deviation of 0.0930. This indicated a low degree of dispersion and relatively concentrated data, meaning that the majority of the data had a small difference from the average. In theory, the kurtosis of the normal distribution was 0, while the kurtosis of this section was − 0.181, suggesting that the data distribution was flatter than normal. However, the difference was not statistically significant. The skewness was 0.012, indicating that the data distribution was approximately symmetrical. The percentile calculations also indicated that the majority of the data was between 0.54 and 0.77, which suggested that the data was relatively concentrated at a similarity level above average. Furthermore, the minimum value was 0.35, while the maximum value was 0.88, indicating the presence of individual data points with significant differences. The data visualization results in Fig. 4A also supported the above analysis results.

### Jaccard coefficient calculation results

Similarly, Table 2 showed the processing results of Jaccard coefficient, and Fig. 4B was a 3D schematic of the original data. The mean value of all Jaccard coefficient was 0.140, indicating a low level of similarity. This implied that the set encompassed a diverse range of elements. The standard deviation was 0.0923, indicating low dispersion, meaning that the majority of the data was relatively close to the mean. However, the kurtosis value of 3.312 indicated that the tail of the data distribution was longer, and compared to a normal distribution, the data had more extreme values. A skewness value of 1.356 indicated that the data distribution was shifted to the left, meaning that most of the data was lower than the average. The percentile results indicated that the range of most data was 0.07–0.32. The minimum value of 0 indicated that there were at least two prescriptions that share few common features with each other. The maximum value of 0.73 indicated that there existed the possibility of a high degree of similarity between individual pairs of prescriptions.

### Comparison of differences between the calculation results of the two metrics

The distributional properties of the data indicated that both the cosine similarity and the Jaccard coefficient exhibited less discrete values. However, the former distribution was closer to a normal distribution, whereas the latter had a longer tail, suggesting the presence of more extreme values. In terms of data range and centroid position, the mean value of cosine similarity was notably high, with the majority of the data range falling within the range of [0.35, 0.88]. This indicated that the method tended to provide moderately high similarity scores. In contrast, the Jaccard coefficient exhibited a lower mean, with the majority of the data falling within the range of [0.07, 0.32]. This indicated that the method tended to provide lower similarity scores.

The main reason for the significant difference in the calculation results between these two methods is their different definitions and calculation methods for similarity. Specifically, the cosine similarity assesses the alignment of the angles or directions of two prescription vectors in a multidimensional space, rather than merely considering the magnitude of the vectors. It is capable of recognizing the impact of elemental alterations on the direction and is more responsive to numerical changes. The Jaccard coefficient, on the other hand, measures the ratio of the intersection and union of two sets of prescriptions, focusing only on the presence or absence of elements, and is therefore less sensitive to numerical changes. While there are some differences in the calculation results of the two indicators, their respective data distribution trends are consistent. In other words, when a value is at a high level of cosine similarity, it is also at a high level of Jaccard coefficient. The results of the two calculations are not mutually exclusive.

To enhance the observation of prescription pairs with higher levels of similarity, the results of the calculations need to be filtered. A threshold value of 0.77 was determined based on the P95 value for the cosine similarity, enabling us to isolate the prescription pairs with notably higher similarity levels. As for the Jaccard coefficient, given that its P95 value of 0.32 did not indicate a high level of similarity, it was determined that 0.5 would be appropriate for screening prescription pairs.

### Prescription pairs with high cosine similarity

A threshold of 0.77 was determined based on the distribution of data, and a total of 68 prescription pairs with high similarity were identified. For the research purpose of this paper, further screening was performed on these 68 prescription pairs to obtain 12 prescription pairs. The steps were as follows: (1) Considering the universality of later experiments, the prescriptions related to pediatric diseases in the results were deleted; (2) To avoid false positive results, the prescriptions that only treat respiratory diseases such as cough and asthma were deleted. Following further processing, the resulting prescription pairs are presented in Table 3. A total of five target prescriptions were used as candidate prescriptions. The prescriptions that match well with the target prescription “Biaoshiganmao Keli” include Biyan Pian (for treating rhinitis-related diseases), Mahuang Tang (for treating neurological and facial diseases in addition to respiratory diseases), and Mahuangjiazhu Tang (for treating rheumatoid arthritis and tinea pedis in addition to respiratory diseases). Prescriptions with a high degree of match to the target prescription “Fenghankesou Keli” include Biyan Pian and Wenge Tang (which can be used to treat diabetes mellitus). A suitable candidate for the target prescription “Kugan Keli” is Gegen Tang. In addition to respiratory diseases, it can also be employed to treat diseases of the musculoskeletal system and the five senses system. Prescriptions with a high degree of match to the target prescription “Tongxuanlifei Wan” include Biyan Pian, Fangfeng Tang (used in the treatment of rheumatoid arthritis and facial neuritis), Mahuang Tang, and Mahuangjiazhu Tang. The prescriptions with high compatibility with the target prescription “Xiaoqinglong Keli” include Mahuang Tang and Mahuangjiazhu Tang.

**Table 3:** 12 prescription pairs with high cosine similarity

No	Target prescription	Prescription to be matched	Cosine similarity
1	Biaoshiganmao Keli	Biyan Pian	0.78
2	Biaoshiganmao Keli	Mahuang Tang	0.77
3	Biaoshiganmao Keli	Mahuangjiazhu Tang	0.77
4	Fenghankesou Keli	Biyan Pian	0.77
5	Fenghankesou Keli	Wenge Tang	0.77
6	Kugan Keli	Gegen Tang	0.79
7	Tongxuanlifei Wan	Biyan Pian	0.81
8	Tongxuanlifei Wan	Fangfeng Tang	0.77
9	Tongxuanlifei Wan	Mahuangjiazhu Tang	0.78
10	Tongxuanlifei Wan	Mahuang Tang	0.78
11	Xiaoqinglong Keli	Mahuangjiazhu Tang	0.78
12	Xiaoqinglong Keli	Mahuang Tang	0.78

### Prescription pairs with high Jaccard coefficient

With a Jaccard coefficient of 0.5 as the threshold, a total of 5 prescription pairs with high similarity were screened, as shown in Table 4. However, further analysis revealed that these 5 prescription pairs had the following issues that made them unsuitable for the purpose of this paper. On the one hand, almost all of the prescriptions were mainly used to treat respiratory diseases related to cough and asthma. On the other hand, from the perspective of prescription composition, the target prescription contained the vast majority, or even all, of the herbs in the prescription to be matched. Such information neither reflects the performance of the calculation method nor leads to new discoveries. Consequently, the Jaccard coefficient calculations presented in this section lack sufficient statistical significance for subsequent analysis. It is possible that this may be related to the dataset constructed in this paper. Should the information in the dataset be expanded, the calculation results may improve, potentially leading to the discovery of new prescription pairs.

**Table 4:** 5 prescription pairs with high Jaccard coefficient

No	Target prescription	Prescription to be matched	Jaccard coefficient
1	Kechuanning Koufuye	Wuhu Tang	0.50
2	Xiaoqinglong Keli	Xiaoqinglong Tang	0.68
3	Xiaoqinglong Keli	Xiaoqinglongjiashigao Tang	0.59
4	Zhikedingchuan Koufuye	Mahuangxingrengancaoshigao Tang	0.67
5	Zhikedingchuan Koufuye	Wuhu Tang	0.73

### Selection of hyperparameters

The model discussed in this paper examined two hyperparameters: the number of hidden units and the number of epochs. The number of hidden units plays a crucial role in processing input data and extracting features, influencing the neural network’s ability to represent the input data. Meanwhile, the number of epochs, which represents the iterations the model undergoes over the entire dataset, can significantly impact the model’s convergence and generalization capabilities. Given the unlabeled nature of the data used in this paper, which implies the infeasibility of manually labeling the similarity values between each prescription node, conventional performance indicators are not suitable for evaluating the model’s performance across different hyperparameters. Consequently, this study opted to determine the optimal hyperparameter values by observing the stability of the model’s output results under various hyperparameter configurations. This approach aims to ensure that the selected hyperparameters enable the model to exhibit robust stability and reliability, even in scenarios where performance cannot be directly evaluated.

**Fig. 5:**
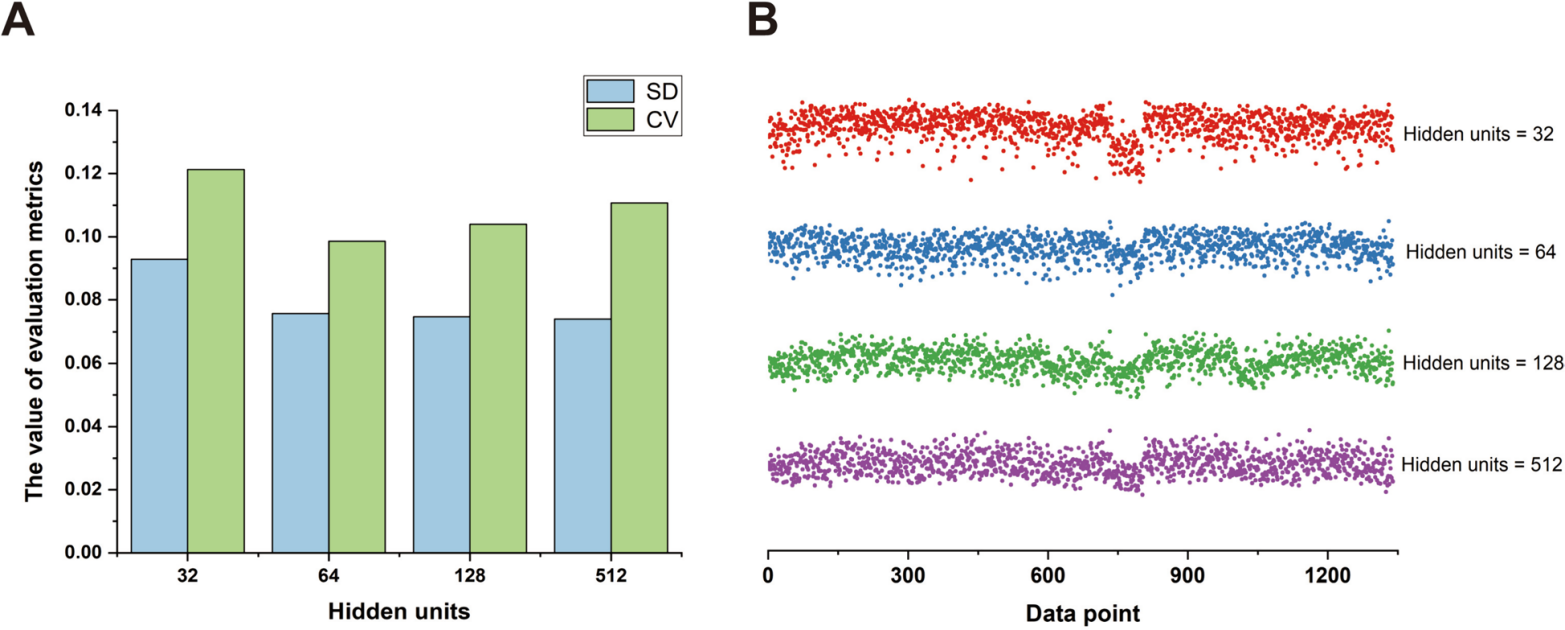
The performance of the model under different numbers of hidden units

While keeping other parameters constant, the number of hidden units was set to 32, 64, 128, and 512, respectively, to calculate the output results of the model under these parameters. The dispersion and variability of the data were measured using the standard deviation (SD) and coefficient of variation (CV). Figure 5A illustrated that, for the standard deviation, the smallest value was observed when the number of hidden units was 128, followed by 64 hidden units, then 512 hidden units, and the largest standard deviation occurred when the number of hidden units was 32. In terms of the coefficient of variation, the minimum value was achieved when the number of hidden units was 64, followed by 128 hidden units, then 512 hidden units, and the maximum coefficient of variation was observed when the number of hidden units was 32. To provide a more intuitive display of the data distribution trends under different numbers of hidden units, Fig. 5B showed the numerical distribution of 1340 data points in each subset. Through comparative analysis, it is evident that when the number of hidden units was 128 and 512, the data distribution exhibited high stability and consistency. Considering both the expressiveness of the calculation results and the complexity of the data used, setting the number of hidden units to 128 was deemed the most appropriate choice.

**Fig. 6:**
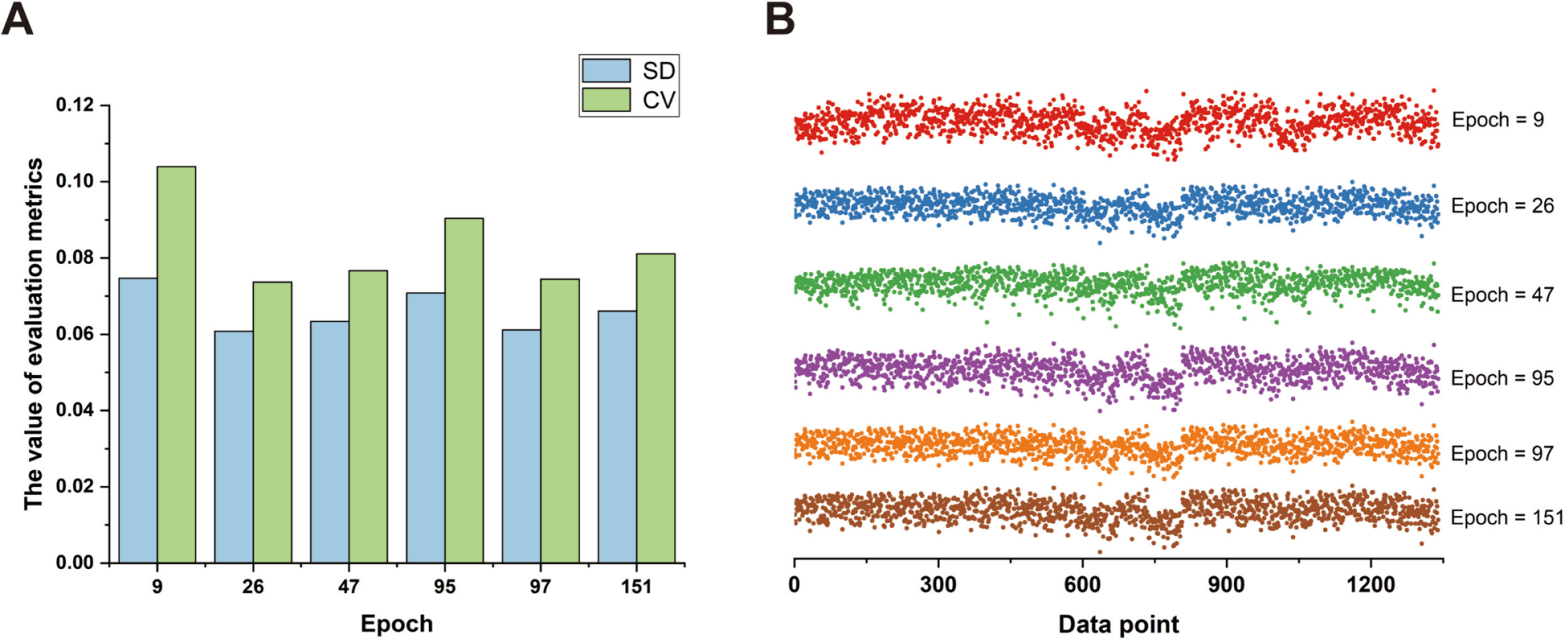
The performance of the model under different numbers of epochs

To evaluate the performance of the model under different parameters, the number of epochs was set to 9, 26, 47, 95, 97, and 151, while keeping other parameters constant. Figure 6A showed that the standard deviation was smallest when the number of epochs was 26, and it gradually increased as the number of epochs changed to 97, 47, 151, 95, and 9, respectively. Similarly, for the coefficient of variation, it was also the smallest when the number of epochs was 26, and the ranking of the coefficient of variation for the remaining epochs was the same as that of the standard deviation. Furthermore, Fig. 6B displayed the distribution of 1340 data points generated by the model under different numbers of epochs. It is evident that when the number of epochs exceeded 26, the output results of the model tended to stabilize. Taking all factors into consideration, 26 was determined to be the optimal number of epochs to maintain the stability of the model.

## Discussion

The conventional approach to drug development has long cycles, exorbitant costs, and a high failure rate in the later stages, which are inadequate to meet the current human health needs. In light of these limitations, the strategy of drug repositioning, which involves the expansion of the existing indications for existing drugs, has emerged as a promising approach to enhance the efficiency of drug development. Moreover, the constant updating of modern testing methods has accumulated a large amount of medical data waiting to be mined, and the development of information technologies such as artificial intelligence has provided more efficient computing methods. These further promote the research progress of drug repositioning. Given the simple composition and clear structure of chemical monomeric drugs, the repositioning research of such drugs has been relatively smoother and has achieved many results. The difficulty in elucidating the medicinal components and efficacy of TCM prescriptions, which are collections of herbs, hinders the calculation of the association between herbs and diseases and greatly hinders research in the field of TCM repositioning. In this paper, based on the hypothesis that similar drugs can treat the same disease, we identified prescriptions with a high match to the target prescription and used the therapeutic use of the matched prescription as potential new indications for that target prescription. However, the experimental program in this paper has the following areas for improvement.

In terms of the completeness of the formula data, this paper does not include information on the weighting factors and dosages of the different herbs in the formula, which is a drawback that needs to be improved. At the outset, we considered this information, but in practice, we encountered some difficulties that could not be resolved immediately. With regard to dosage, it should be noted that this paper concerns the ancient prescription of Chinese medicine and that the unit of herb dosage recorded in the ancient prescription is not consistent with the unit of modern dosage. Furthermore, the unit of dosage for some herbs is not routinely used in practice. Consequently, this irregular information is not suitable to be added into the dataset. Concerning the weighting factor, when selecting herbs for a TCM prescription, there is a principle of “sovereign, minister, assistant, and courier”. This implies that different herbs contribute to the prescription in varying degrees. However, upon attempting to assign coefficients to herbs, it became evident that there is a dearth of authoritative and recognized reference material on the weighting coefficients of herbs. The introduction of subjective error was deemed unacceptable, and thus the decision was made not to include information on weight coefficients. However, to ensure more accurate and reliable calculation results, it is necessary to consider these two factors. This is also a common research problem in the field of TCM data mining at present.

Regarding the similarity calculation results in this paper, there are some significant differences between the two methods chosen, and the reasons for this have also been analyzed above. In terms of data availability, the result of cosine similarity has more value for subsequent use. Given that the existing dataset still requires further enrichment, the next stage of the project will involve the collection of real-world electronic medical records based on herbs. These will be used to supplement the original graph structure data with additional node and edge information. Furthermore, if the quantity of data in the dataset increases, it would be possible to construct a recommendation system based on GCN to identify new indications for the target prescriptions and compare the results with those presented in this paper. In the later stages of the research process, a specific target prescription can be selected as the development object, combined with network pharmacology and pharmacological efficacy experiments for verification.

## Conclusions

In conclusion, this paper presents a method based on self-supervised graph convolutional neural network for calculating the similarity between prescriptions and identifying new indications. Additionally, the paper compares the differences in the results of the two calculation indicators. This method is helpful in predicting the new treatment approaches of Chinese patent drugs that have been marketed, improving the clinical value of Chinese patent drugs, and further promoting their secondary development. The follow-up work will focus on enriching the content of the dataset and adding more similarity measurement indicators, such as the diffusion and proximity algorithms, to increase the accuracy of the results. We hope that the method proposed in this paper will serve as a valuable reference point for the field of traditional Chinese medicine repositioning.

## Supplementary Information

Additional file 1

## Data Availability

The datasets used and/or analyzed among the current study are available from the corresponding author on reasonable request.

## Abbreviations

DGI: Deep graph infomax
GCN: Graph convolutional network
GNN: Graph neural network
TCM: Traditional Chinese medicine
